# Editorial: Human and Animal Models for Translational Research on Neurodegeneration: Challenges and Opportunities From South America

**DOI:** 10.3389/fnagi.2018.00095

**Published:** 2018-04-06

**Authors:** Agustín Ibáñez, Lucas Sedeño, Adolfo M. García, Robert M. J. Deacon, Patricia Cogram

**Affiliations:** ^1^Laboratory of Experimental Psychology and Neuroscience (LPEN), Institute of Cognitive and Translational Neuroscience (INCYT), INECO Foundation, Favaloro University, Buenos Aires, Argentina; ^2^National Scientific and Technical Research Council (CONICET), Buenos Aires, Argentina; ^3^Universidad Autónoma del Caribe, Barranquilla, Colombia; ^4^Center for Social and Cognitive Neuroscience (CSCN), School of Psychology, Universidad Adolfo Ibáñez, Santiago, Chile; ^5^Centre of Excellence in Cognition and its Disorders, Australian Research Council (ACR), Sydney, NSW, Australia; ^6^Faculty of Education, National University of Cuyo (UNCuyo), Mendoza, Argentina; ^7^Faculty of Science, Institute of Ecology and Biodiversity, University of Chile, Santiago, Chile

**Keywords:** neurodegenerative diseases, South America, research, clinical protocols, neurosciences, public health, animals, human experimentation

## Neurodegeneration: a voice from the South

Facing the alarming growth of dementia and neurodegenerative conditions has become a critical priority across the globe (Alzheimer's Disease International, 2009; Lancet, 2015; Shah et al., 2016; Parra et al., 2018). Neurodegenerative diseases are the most frequent cause of dementia, representing a burden for public health systems (especially in middle and middle-high income countries). Although most research on this subject is concentrated in first-world centers, growing efforts in South American countries (SACs) are affording important breakthroughs. This emerging agenda poses not only new challenges for the region, but also new opportunities for the field at large. SACs have witnessed a promising development of relevant research in humans and animals, giving rise to new regional challenges. As highlighted in a recent experts' consensus paper Latin-American countries (LAC), and SACs in particular (Parra et al., 2018), face a critical situation. Higher demographic rates and the predicted prevalence of dementia have reached and even exceeded those of developing countries. In SACs, low- and middle-income countries (e.g., Bolivia, Paraguay), the prevalence of dementia will double that of high-income countries, while upper-middle-income countries in the region (e.g., Argentina, Brazil, Chile, Colombia, Peru, Uruguay, and Venezuela) will experience the greatest impact of dementia. The WHO estimated that the standardized prevalence of dementia in Latin America was 8.5%, but multiple SACs have been underrepresented or underestimated in such a calculation (Parra et al., 2018). Moreover, raw prevalence rates across studies are characterized by high variability within and between countries (e.g., Argentina: 8.3; Brazil: 7.1-2.0; Chile: 4.4-7.0; Colombia: 6.0; Peru: 6.72-9.3; Uruguay: 3.1; Venezuela: 5.7-13,7) (Parra et al., 2018). In addition, most of these studies are undermined by various limitations and methodological problems. Even considering these data, SACs possess the highest global prevalence of dementia after North Africa/Middle East in people above the age of 60 (Parra et al., 2018). Moreover, the harmonization of global strategies against dementia in these contexts is hindered not only by reduced epidemiological data, but also by the lack of standardized clinical practice, insufficient training of physicians, limited resources, and poor governmental support, let alone poverty and more general cultural barriers and stigmas. All of these factors have impacted the type and amount of research conducted in SACs. A regional network, based on multi-institutional actors from research, governmental, and private sectors is fundamental to overcome these challenges (Parra et al., 2018).

Nevertheless, until now most research groups still work in isolation or in sporadic collaboration, without developing large-scale multicenter studies or active cooperation networks. The field could grow exponentially by combining the strengths of regional research with higher visibility, a translational philosophy, and enhanced global networking. Importantly, collaborative developments may promote the establishment of translational centers studying neurodegeneration. This Research Topic engages researchers from the world over, helping to integrate the international community of experts and to establish new challenges and developments for future investigation. We present original research in SACs, including studies assessing the interplay among genetic, neural, and behavioral dimensions of these diseases, as well as articles on vulnerability factors, comparisons of findings from various countries, and works promoting multicenter and collaborative networking. More generally, our Research Topic covers a broad scope of human research approaches (behavioral assessment, neuroimaging, electromagnetic techniques, brain connectivity, peripheral measures), animal methodologies (genetics, epigenetics, proteomics, metabolomics, other molecular biology tools), target species (human and non-human animals, sporadic, and genetic versions), and article types (mainly original research articles, but also case reports, data reports, commentaries, opinions, and reviews), all based on work conducted in SACs. Thus, in capturing the breakthroughs, possibilities, and limitations of such a promising niche, the present Research Topic (titled *Human and animal models for translational research on neurodegeneration: challenges and opportunities from South America*) represents a valuable forum to initialize a constructive dialogue and reflect on the present and future of neurodegenerative research in the region. Here, we summarize the main contributions included in the volume. Through this wide-ranging proposal, we hope to introduce a fresh approach to the challenges and opportunities of research on neurodegeneration across these countries, focusing on two overarching levels of evidence (human and animal research), as summarized below.

## Studies on humans

Concerning human research, SACs offer invaluable possibilities to pursue neurogenetic studies and clinical trials. This region possesses the world's largest population of familial Alzheimer's and Huntington's disease (AD and HD, respectively), among others, alongside multiple novel and rare functional genomic variants of other disorders. Moreover, poor socioeconomic conditions in several communities provide a natural scenario to study the role of vulnerability, resilience, and genetic-cultural interaction on disease progression. These opportunities are already being exploited by consolidated research groups in Argentina, Chile, Colombia, Peru, and Brazil, among others, via cutting-edge approaches which include connectomics, omics-biomarkers, and neuropsychological assessment. Moreover, the emerging cognitive neuroscience of neurodegeneration in the region (Parra et al., 2010, 2015; Ibanez and Manes, 2012; Baez et al., 2013, 2014a,b, 2015, 2016a,b, 2017; García-Cordero et al., 2015, 2016; Melloni et al., 2015, 2016; Pietto et al., 2016; Santamaría-García et al., 2016, 2017; Sedeño et al., 2016, 2017; Abrevaya et al., 2017; Birba et al., 2017; Calvo et al., 2017; Dottori et al., 2017; Garcia et al., 2017a; García et al., 2017b,c; Ibáñez et al., 2017; García and Ibáñez, 2018; Kumfor et al., 2018) has provided manifold pathways of synergy with multimethodological approaches to genetic, clinic, neuropsychological, and neuroscientific data.

The research on human neurodegeneration presented in this collection includes works on: (a) global challenges to dementia, from diagnosis to public health; (b) different dimensions of assessment (low socio-educational levels, cultural and competence-related variability, and robust evaluations to face heterogeneous contexts); (c) neurodegeneration discrimination and disease progression (through combinations of behavioral measures, neuroscientific approaches, and biomarkers for improving differential diagnosis between dementia subtypes), the characterization of specific initial alterations in each disease, and the identification of factors that manifest in early aging; and (d) the impact of non-pharmacological interventions for dementia using non-invasive brain stimulation.

### Global challenges and public health

A first group of studies assesses diverse aspects of the global challenges related to dementia, from diagnosis to public health at a regional level. After reviewing critical sociodemographic and epidemiological data form LACs, Baez and Ibanez propose to evaluate the plausibility of international expert recommendations regarding dementias in these countries. Key issues of this evaluation include diagnosis, demographic specificities of LACs, lack of social awareness of these diseases, deficiencies in the health system, the need for standardizing diagnostic practices, and the existing barriers in terms of resources and cultural factors. Similarly, Custodio et al. outline a challenging picture of epidemiological data in LACs, evidencing the major impact of unprecedented demographic changes and projections of dementia for people between 65 and 69 years old. The situation is worst for low-income people whose families cover the majority of the cost related to the disease. This is even worse for illiterate people, where the majority of the costs are covered by families. Accordingly, the authors propose a critical assessment of regional differences and similarities for the implementation of long-term care policies and plans. For their own part, Cardona-Gómez and Lopera assess the intertwine of novel animal and human translational research on molecular targets and pre/clinical studies. Then they discuss on cases of pure and mixed dementias in the region, and, finally, they recommend the implementation of a protocol clarification policy for developing clinical trials and local intervention strategies.

### Multiple and multimodal assessment

Another set of articles focuses on various dimensions of assessment, including the effects of low socioeconomic status and educational level, the role of clinical competence, and the relevance of trans-culturally valid tasks. The low detection of dementia is a major problem in SACs, accentuated by the lack of validated and standardized tools. In a cross-sectional study, Custodio et al. evaluate the robustness of the memory alteration test (MAT) in low-educated patients with mild cognitive impairment (MCI) and AD by using validity measures (sensitivity, specificity, and correctly classified percentage), internal consistency (Cronbach's alpha coefficient), and concurrent validity (Pearson's ratio coefficient between the MAT and Clinical Dementia Rating scores). All measures, they conclude, provide robust and adequate classification scores.

Some instruments, like the working memory binding (WMB) task (Parra et al., 2009, 2010), seem to have high sensitivity and specificity to detect AD in early stages. It has been suggested that cultural aspects such as education, age, and memory abilities may impact in relatively culture-free tasks (Parra et al., 2018). Building on this line of research, Hoefeijzers et al. provide evidence of a new WMB task composed by everyday items that is not affected by education. This result suggests that the WMB test may be culturally and educationally unbiased for the screening of abnormal aging trajectories.

### Neurodegenerative diseases: progression and differentiation

A third set of works integrates behavioral and neuroscientific insights (including biomarker research) to examine differential diagnosis across dementia subtypes, the characterization of specific initial alterations for each disease, and the identification of factors that impact early aging.

Russo et al. investigate whether memory recognition and deferred recall measures of episodic memory, in combination with cerebrospinal fluid (CSF) biomarkers, can predict the conversion from MCI (397 amnestic-MCI patients of the Alzheimer's disease Neuroimaging Initiative, ADNI) to AD (at 24 months of follow-up). Predictive models of memory, together with risk factors (age, sex, education, APOE genotype and CSF biomarkers), evidence that memory measures alongside amyloid biomarkers can predict the conversion of MCI to AD in the ADNI cohort, especially when combined with amyloid biomarkers. This highlights the relevance of a multimodal approach to anticipate the MCI progression to dementia.

Guevara et al. introduce an ante-mortem method to differentiate progressive supranuclear palsy (PSP) from idiopathic Parkinson's disease (IPD) at early stages. To this end, the authors combine normalized measures of brain atrophy with clinical metrics (Unified Parkinson's Disease Rating Scale Part III, Hoehn and Yahr, Clinical Global Impression for Disease Severity Scale, and the Frontal Assessment Battery). Their results show that whole-brain and gray matter volumes distinguished PSP from IPD, and that clinical-imaging correlations were indicative of clinical presentation and differentiation.

Campêlo et al. investigate the relationship among single nucleotide polymorphisms of alpha-synuclein gene (SNCA) and risk for PD in a Brazilian sample, considering potential interactions with environmental factors and specific clinical outcomes (cognitive, motor, and mood impairments). Their findings confirm the association between SNCA and PD risk (and early onset PD). Specific SNCA alleles were significantly more frequent in PD patients with cognitive impairment, and negative association with protective factors (cognitive activity and smoking habits). This study constitutes the first description of SNCA polymorphism and PD in a South-American sample.

The relation among familial antecedents of late-onset AD (LOAD), cognitive impairment, and sleep patterns in asymptomatic subjects was investigated by Abulafia et al. Middle-aged children of patients with LOAD (O-LOAD), in comparison with controls, displayed deficits in episodic memory and language. Moreover, the former group showed a phase-delayed rhythm of body temperature. Also, cognitive performance in these subjects was associated with cardiac autonomic sleep-wake variables (greater sympathetic activity at night was related to worse cognitive performance).

Long-lasting neurofunctional influences during childhood can impact pathological aging. Iron deficiency anemia (IDA) is a marker of iron micronutrient deficit affecting myelination, dopamine neurotransmission, and neuronal metabolism. Algarin et al. explore the connection between IDA in infancy and altered connectivity patterns of the default-mode network (DMN, an aging-sensitive resting-state network) in young adults. Compared to controls, participants with IDA evidenced atypical DMN connectivity. These preliminary findings suggest that a common nutritional problem among human infants may be important for understanding aberrant aging mechanisms.

Senescence has been associated with metabolic changes including mitochondrial fission and fusion events. Stab et al. assess the cell senescence and structural remodeling of mesenchymal stromal/stem cells (from human mitochondrial tissue) isolated from adipose tissue *in vitro*. Cell morphology aging was associated with an increase in β-galactosidase activity. Old cells showed increased mitochondrial mass, augmented superoxide production, and decreased mitochondrial membrane potential. Morphological changes were related to increases in mitochondrial fusion proteins, Mitofusion 1, and Dynamin-related GTPase. Thus, aged, adipose tissue-derived mitochondrial stem cells developed a senescent phenotype.

### Non-invasive brain stimulation as non-pharmacological interventions

A fourth set of studies evaluate the impact of non-pharmacological interventions for dementia. Non-invasive brain stimulation (NIBS) methods can induce plastic changes in the brain and modulate cognitive functions in humans. Thus, their potential use for early stages of dementia has become a promissory strategy.

Birba et al. conducted a systematic review of the effects of NIBS on MCI and subjective cognitive impairment (SCI) in preventing or delaying the development of AD. In particular, they discuss the impact of NIBS on specific target functions, including recognition of verbal and non-verbal stimuli, attention, psychomotor speed, and everyday memory. Moreover, they identify a number of methodological issues (differences among tasks, designs, and samples size) that arguably underlie the mixed results obtained so far. They also outline further methodological approaches to boost the efficacy and specificity of NIBS in MCI and SCI. These issues are critical for developing robust treatments for both conditions.

Finally, another report by Birba et al. provides the first evidence that direct electrical brain stimulation can enhance performance in the working memory binding (WMB) task, a sensitive tool for early AD. WMB deficits constitute a robust clinical and preclinical marker of AD, associated with early atrophy of posterior brain regions. Profiting from a unique approach, the authors show that direct intracranial electrical stimulation of the parietal cortex can induce a selective improvement in WMB performance. These preliminary but promising results promote new opportunities to improve binding functions in preclinical AD through brain stimulation.

## Studies on animals

The region also constitutes a rich platform for developments via animal research. Preclinical testing of new therapeutic concepts has been difficult due to the lack of naturally occurring disease models. Although availability of genetically engineered mouse models has partly addressed this challenge, neurodegenerative diseases rarely occur in non-human animals (Jucker, 2010); and the causes of non-familial dementia are multifactorial and age-related (Hurley et al., 2018). In consequence, non-genetic and natural models of dementia are still required. Advances could be made by studying species such as the *Octodon degus*, an endemic rodent from Chile that spontaneously develops an analog of dementia at behavioral and neurobiological levels (Ardiles et al., 2012; Hurley et al., 2018). In addition, the *O. degus* can also naturally develop several other conditions like diabetes mellitus type 2, macular and retinal degeneration and atherosclerosis, conditions that are often associated with aging and comorbid disorders with dementia (Hurley et al., 2018). Consequently, the *O. degus* is a suitable novel experimental model that can be utilized for the development of disease-modifying treatments for dementia. In addition, scientific efforts in the region have been enhanced through new models from promising groups (e.g., single and double immunohistochemistry, inmunoblot, RT-PCR, behavioral phenotyping, CNS lesioning techniques, neuronal tracers for connectivity studies, small craniotomy for drug delivery). These approaches are now fueled by the engagement of international centers and the development of multicenter alliances.

The section on animal research provides timely works on regionally relevant topics. These include (a) natural models of AD; (b) pathophysiological models of different neurodegenerative conditions, such as frontotemporal dementia (FTD), amyotrophic lateral sclerosis (ALS), and AD; (c) the role of the proteostasis network in basic organisms; and (d) cognitive interventions as early non-pharmacological therapeutics.

### Natural rodent models of neurodegeneration

In a *Data Report Article*, Altimiras et al. offer the first characterization of the *O. degus*'s brain transcriptome (in order to support its use as a natural model of AD), together with a comparison between the transcriptomes of AD-like and healthy specimens. Of note, this work includes an unprecedented report of whole transcriptome sequencing (RNA-seq) of the *Octogon degus* brain. Results reveal differences in novel and previously reported genes for AD and related disorders (CHRNA6, AMD1, WISP1, COX8A, APOC-I, among others). In addition, the comparison of human and *O. degus* AD-like brain transcriptomes evidences multiple common genes in both species. These findings highlight the relevance of this rodent species to foster progress in AD research.

Braidy et al. use the *O. degus* model to evaluate the biometal imaging and role of metal uptake transporters in AD pathogenesis and aging. Their work hinges on the hypothesis that *O. degus* may develop neuropathological abnormalities in the distribution of redox active biometals, due to alterations in the expression of lysosomal protein, major Fe/Cu transporters, and selected Zn transporters (ZnTs and ZIPs). Using laser ablation inductively coupled plasma mass spectrometry, they find elevated quantitative images of biometals (Fe, Ca, Zn, Cu, and Al) in the aged *O. degus*, which in turn showed an age-dependent rise. Some of these metals were specifically enriched in the cortex and hippocampus. Whole-brain extracts evidenced age-related deregulation of metal trafficking pathways (impaired lysosomal function, demonstrated by increased cathepsin D protein expression). An age-related reduction in the expression of subunit B2 of V-ATPase was also identified, alongside significant increases in amyloid beta peptide 42 (Aβ42), and metal transporter ATP13a2. Finally, enhanced expression of transporter of divalent metal species, 5′-aminolevulinate synthase 2 (ALAS2), and the proto-oncogene, FOS was associated with aging. Thus, these results suggest that transition metals in the brain may be enriched with age in the *O. degus*, and that metal dyshomeostasis in specific brain regions may be related to age.

### Pathophysiological models of different neurodegenerative conditions

Two additional studies provide pathophysiological models of FTD, ALS, and AD. FTD and ALS are both associated with TAR DNA-binding protein 43 (TDP-43). To investigate the behavioral phenotype associated with this proteinopathy, Alfieri et al. implemented a transgenic (Tg) mouse model that conditionally overexpresses human wild-type TDP 43 protein (hTDP-43-WT) in forebrain neurons. They analyzed the motor, social, and cognitive performance of this species. The young hTDP-43-WT Tg mice presented a mild degree of spasticity. Analysis of social and cognitive behavior showed a rapid installment of deficits in social interaction, working memory, and recognition memory. After long-term (up to 12 months) transgene induction, a motor phenotype (previously absent in younger mice) was identified. Thus, this work points to the time-dependent emergence of a motor phenotype, a clinical presentation of FTD with involving motor deficits, and a complementary animal model for studying TDP-43 proteinopathies.

The effect of chronic treatment with reserpine (an inhibitor of vesicular monoamine transporter-2), which induces dyskinesia in PD, has been proposed to be attenuated in spontaneously hypertensive rats (SHRs). Leão et al. evaluated whether SHRs (in comparison with Wistar rats) present differential susceptibility to repeated reserpine-induced deficits in a progressive model of PD. Only reserpine-treated Wistar rats presented increased motor signs. After a withdrawal period, both strains recovered from motor impairment, but SHRs were slower to reach control levels. Immunohistochemistry for tyrosine hydroxylase (TH) and α-synuclein (α-syn) after the last injection or 15 days after withdrawal showed a reduction in TH and an increase in α-syn immunoreactivity in the substantia nigra and dorsal striatum (recovered after 15 days of withdrawal). The SHRs were resistant to reserpine-induced TH decrement in the substantia nigra, and presented reduced immunoreactivity to α-syn in the dorsal striatum relative to Wistar rats, irrespective of treatment. In brief, SHRs may be resilient to motor and neurochemical impairments induced by the repeated low-dose reserpine.

Accumulation of β in AD begins many years before clinical onset. Yet, given that massive accumulation of Aβ appears in 30% of healthy aged individuals, compensatory mechanisms and/or additional neurotoxic or protective factors need to be discovered. Belfiori-Carrasco et al. provide a novel genetic screen in the drosophila brain that identifies modifiers of age-dependent amyloid β toxicity. One hundred and ninety-nine deficiency lines accounting for ~6,300 genes were analyzed. Six lines significantly modified Aβ42 neurotoxicity, including the CG11796 and CG17249 (orthologs to human HPD and PRCC, respectively) as candidates. These modifiers of Aβ42 neurotoxicity in Drosophila open avenues for new validation studies into their possible role in sporadic AD.

### Proteostasis network: from animal to human approaches

Abnormal protein aggregation is a transversal pathological mechanism in neurodegeneration. Thus, the capacity of neurons to handle alterations in the proteome seems to be specifically altered in aging. Martínez et al. critically asses the proteostasis network in basic organisms, highlighting the challenges for moving toward human research in this domain. Although several reports are pointing to this network as a relevant adjustor of organismal aging in several species, its relevance to human aging remains unknown. The authors discuss multiples challenges (regarding buffering capacity, neural control of organismal proteostasis, connections among stress and aging in protein misfolding disorders, control the cell-nonautonomous UPR as a therapeutic strategy) in the light of new drug-based avenues to intervene in brain aging.

### Cognitive interventions as an early non-pharmacological strategy: from animal research to human translation

To conclude, Gehres et al. set forth a challenging opinion on cognitive interventions as an early non-pharmacological strategy in AD, considering a translational perspective from animal research to human behavior. The critical concepts of cognitive reserve, cognitive interventions, and early-life exposure to environmental enrichment (EE) are reviewed from the vantage point of animal research, with new vistas for neurodegenerative human conditions. After an informative revision, the authors conclude that study designs that aim to unravel EE-specific mechanisms are crucial and could guide the generation of non-pharmacological strategies. Moreover, the combination of EE with better models of sporadic AD, in conjunction with CSF/PET biomarkers, could promote novel insights on novel therapeutic targets for AD.

## Conclusions

Amid a multiple collection of theories, experimental approaches, and models, all these studies highlight both the rise of world-class research in SACs, as well as the specificity of problems and opportunities in the region. Moreover, they provide relevant evidence for the harmonization of multilevel approaches to neurodegenerative research. Overall, this integrated and pluralistic approach to neurodegeneration in SACs can provide the basic building blocks for a future translational network based on (but not limited to) experimental research, focusing on policy changes and the development of international collaborations (Parra et al., 2018). In sum, this book demonstrates that SACs are highly active in generating first-class translational and multicenter research on neurodegeration, thus amplifying the powerful voice of the South in this worldwide program.

## Author contributions

AI: designed the proposal. AI, LS, and AG: wrote the first draft, discussed contributions from all co-authors, and approved the final version. All authors (AI, LS, AG, RD, and PC) searched the literature, participated in discussing the contents of the paper, contributed to editing and approved the final version of the article.

### Conflict of interest statement

The authors declare that the research was conducted in the absence of any commercial or financial relationships that could be construed as a potential conflict of interest.
